# Time-course of muscular stress and fatigue markers following different soccer small-sided games: evidence from a randomized crossover study

**DOI:** 10.3389/fpubh.2025.1767991

**Published:** 2026-01-16

**Authors:** Xiaoshuang Wang, Yongxing Zhao, Yuqing Duan, Qiling Qiu

**Affiliations:** 1 College of Physical Education, Chizhou University, Chizhou, Anhui, China; 2 College of Sports Science, Hefei Normal University, Hefei, Anhui, China; 3 School of Physical Education, Anhui Normal University, Wuhu, China

**Keywords:** football, injury risk, muscle fatigue, muscle readiness, muscle recovery, sports performance

## Abstract

**Introduction:**

High-intensity exercises with substantial mechanical demands can contribute to greater muscular loading, potentially increasing fatigue and potentially increasing fatigue and requiring careful load-management for preventing injury and enhancing recovery planning. This study aimed to compare the effects of 2v2, 4v4, and 6v6 small-sided games (SSG) formats on muscle stiffness, creatine kinase levels, and reactive strength index (RSI) immediately post-exercise and 24 hours thereafter.

**Methods:**

A randomized crossover design evaluated 36 male under-23 players from two regional-level amateur football teams. Participants completed 2v2, 4v4, and 6v6 over three weeks in a counterbalanced sequence, with assessments at baseline, immediately after, and 24 hours post-session. Rating of perceived exertion (RPE) was always assessed after SSGs, while muscle stiffness, creatine kinase and RSI were measured in all the time points.

**Results:**

Muscle stiffness was higher in 2v2 than 4v4 (MD = 21.722, p < .001) and 6v6 (MD = 19.514, *p* = .002) at 24h (*F* = 9.346, *p* < .001, ηp^2^ = .151). Creatine kinase was greater in 2v2 across both time points (all *p* < .001), while RSI was lowest in 2v2 (*F* = 29.313, *p* < .001, ηp^2^ = .358). RPE was significantly highest in 2v2 (*F* = 42.490, *p* < .001), and showed strong correlations with CK (*r* > .63, *p* < .001), moderate with stiffness (*r* = .314), and negative with RSI (*r* = –.344, *p* < .001).

**Conclusions:**

The results suggest that 2v2 games lead to significantly higher muscle stiffness and perceived exertion, while eliciting higher CK levels and lower RSI compared to 4v4 and 6v6 formats. This suggests that small-sided games impose greater neuromuscular and muscular stress, likely due to the higher intensity typical of these formats. Coaches may therefore consider these demands when managing training loads and when planning recovery to potentially reduce excessive residual fatigue and support readiness.

## 1 Introduction

Small-sided games (SSGs) are widely used in soccer training due to their ability to simultaneously develop physical, technical, and tactical aspects of the game (1–4). These games allow coaches to work on specific tactical principles and inter-sector player articulation while replicating match-like situations (5). The effectiveness of SSGs can be manipulated by adjusting factors such as field dimensions, number of players, and task objectives, which influence physiological and technical variables (6). Research has shown that SSGs are as effective as interval training in maintaining aerobic fitness in young elite soccer players (7, 8). Moreover, players report greater physical enjoyment when participating in SSGs compared to interval training (9).

SSGs in soccer training offer varying physical and physiological demands based on game format and player numbers (10). In this context, physiological load refers to internal load (the athlete’s psychophysiological response, such as heart rate and rating of perceived exertion, RPE), whereas physical demands refer to external load (the mechanical/work output, such as total distance, high-speed running, and accelerations/decelerations) (11). Reducing player numbers increases physiological load but may decrease physical demands in possession play formats (12). SSGs with 2v2, 3v3, and 4v4 formats all provide game-like intensities suitable for aerobic fitness improvement, with 2v2 placing higher demands on anaerobic energy systems (13). On the other hand, larger formats tend to have greater field dimensions, which are associated with increased physical demands, including more running at higher speeds such as high-speed running and sprinting (14). Therefore, when comparing formats, it is important to keep the pitch area per player consistent to minimize confounding effects related to pitch size.

The intensification of physical demands during SSGs can contribute to muscle performance impairments and delayed recovery. For example, the relative playing area in SSGs has been shown to influence hamstring fatigue, with larger areas leading to greater reductions in hamstring force (15). Additionally, the number of accelerations performed during SSGs has been linked to decreased hamstring peak torque (15). SSGs are also associated with prolonged exercise-induced muscle damage, short-term neuromuscular fatigue, and slow recovery kinetics in strength, jump, and sprint performance (16). This is further supported by another study reporting impairments in sprint performance and acceleration following SSGs (17).

Despite existing evidence on performance impairments associated with various SSGs, limited information is available regarding their impact on muscle-level mechanical properties and muscle damage, which are crucial for a comprehensive understanding of muscle functionality. For instance, assessing muscle stiffness can provide insights into acute changes in muscle mechanical behavior following loading, which may accompany fatigue and recovery processes (18). However, stiffness should be interpreted as an indirect, non-specific indicator that can also be influenced by factors such as muscle tone, swelling, passive tension, and temperature (19). Additionally, monitoring creatine kinase levels serves as a pertinent marker for muscle damage and recovery status post-exercise (20). These analyses can complement evaluations of muscle functionality, such as the reactive strength index (RSI), which assesses the efficiency of the stretch-shortening cycle (21). RSI derived from the drop jump reflects fast stretch–shortening-cycle function (achieving high jump height with short ground contact time) and is commonly used to monitor neuromuscular status and shows associations with strength and speed qualities relevant to field sport performance (22, 23).

Although these aspects are relevant for a holistic view of fatigue and recovery induced by SSGs, prior research has typically examined performance outcomes and/or biochemical markers in isolation (17, 24, 25), and has rarely compared muscle mechanical properties (stiffness), CK responses, and RSI within the same experimental protocol across multiple SSG formats and short recovery windows (immediate and 24 h), particularly under conditions where relative area per player is held comparable across formats to reduce pitch-size confounding. The present randomized crossover study addresses this gap by quantifying acute and 24 h responses of stiffness (biceps femoris), CK, and RSI following 2v2, 4v4, and 6v6 SSGs in the same players. Given the varying physical demands of SSGs [ranging from very small formats like 1v1 or 2v2, which differ significantly from larger formats like 4v4 or 5v5 in terms of accumulated distance at various speed thresholds and the number of accelerations and decelerations as reported in previous studies (26)]. Such knowledge is essential to optimize the integration of these games into training regimens and to implement appropriate recovery strategies, thereby mitigating their impact on subsequent training sessions or competitive events. Considering this significance, the aim of the present study was to compare the effects of 2v2, 4v4, and 6v6 SSG formats on muscle stiffness, creatine kinase levels, and RSI immediately post-exercise and 24 h thereafter. Correlation analyses between RPE and remaining markers were also conducted as complementary/exploratory analyses to contextualize the main comparative findings and were not part of the primary inferential objective. These findings are intended to inform format selection and short-term recovery planning in training microcycles by indicating which SSG formats are associated with larger acute and 24 h perturbations in perceptual (RPE), neuromuscular (RSI), and biochemical (CK) markers.

## 2 Methods

### 2.1 Experimental approach to the problem

This study employed a randomized crossover design in which all participants took part in SSGs of three different formats: 2v2, 4v4, and 6v6. The intervention spanned three consecutive weeks during the mid of the season, with each player completing all three formats in a randomized and counterbalanced order to minimize sequencing effects. Each condition was separated by approximately 7 days (weekly microcycle), and players completed their usual team training between study sessions. To minimize week-to-week variability, the SSG sessions were implemented at the same time of day and in the same training slot, and no additional training session was performed on the SSG day. Each week, players participated in only one format. For each SSG format, player assessments were conducted at three time points: baseline (prior to the SSG), immediately after the SSG session, and 24 h post-SSG. The SSG sessions were consistently scheduled to take place 48 h after the players’ last official match, ensuring standardized recovery time across all conditions. Randomization determined the order in which players experienced each format. For example, some players followed a 2v2 → 4v4 → 6v6 sequence, others followed 4v4 → 2v2 → 6v6, and 6v6 → 2v2 → 4v4. Participants were assigned to one of these sequences in a balanced manner by drawing lots from a container, with each sequence written on an equal number of slips of paper. This ensured that all sequences were represented evenly across the sample. Evaluator blinding was maintained throughout data collection; however, participants were aware of the format they were engaging in, due to the nature of the task. Participants were not provided with any specific recovery strategy and were instructed not to use any recovery methods after the intervention. On the day of SSG implementation, no training session was conducted, ensuring that the evaluation of outcomes reflected only the impact of the SSG.

### 2.2 Participants

An *a priori* sample size estimation was conducted using G*Power (version 3.1.9.7) to determine the minimum number of participants required to detect statistically significant differences between SSG formats across time points in a crossover design. The analysis was based on a repeated-measures ANOVA model with two within-subject factors: format (three levels: 2v2, 4v4, 6v6) and time (three levels: baseline, immediately post-SSG, and 24 h post-SSG). Assuming a small-to-moderate effect size (*f* = 0.2), *α* = 0.05, power = 0.80, correlation among repeated measures *r* = 0.5, and nonsphericity correction *ε* = 0.75, the required sample size was estimated at 28 participants.

Participants were recruited using a convenience sampling strategy from two local amateur soccer teams competing at the regional level. These teams were selected based on their availability, willingness to participate, and regular engagement in SSGs. Participants were male under-23 (U23) regional-level amateur football players, therefore, findings should be interpreted within this target population. Players from both teams were allocated across the crossover sequences in a balanced manner to avoid team clustering within any sequence. Eligibility required being injury-free at the time of data collection and without a time-loss lower-limb injury in the preceding 4 weeks. The recruitment process involved direct communication with team managers, who provided initial consent for team participation. Players were then invited to join the study if they met the inclusion criteria, which included being injury-free and capable of participating in the physical demands of the study. Each player was individually briefed on the study’s purpose, procedures, and potential risks, and voluntary written informed consent was obtained prior to participation.

Ethical approval for the study was obtained from the institutional review board (Hefei Normal University) with the code number 2024LLSP007. All participants provided voluntary informed consent before their involvement in the study. The study adhered to the principles of the Declaration of Helsinki for ethical conduct in research involving human participants. Participants were also informed that the research involved no significant risks beyond those encountered in normal training or competition, and appropriate safety measures were implemented.

The participants in this study were 36 male under-23 players from two regional-level amateur football teams. The players, with an average age of 20.1 ± 0.7 and training experience of 7.9 years, were involved in 3 to 4 training sessions per week, each lasting approximately 60 to 90 min. Training sessions included a combination of fitness drills, technical skill development, and tactical exercises, as well as match-specific preparations. Players typically participated in one competitive matches per week.

### 2.3 Small-sided games

All SSGs were conducted at the beginning of the regular training schedule, starting at 5:00 p.m., on a synthetic turf under environmental conditions of 19.3 °C ± 2.1 °C and 61.3% ± 3.2% relative humidity. Prior to the SSGs, a standardized warm-up protocol was implemented by the same researcher. This warm-up consisted of 7 min of jogging, 8 min of dynamic stretching focusing on the quadriceps, hamstrings, adductors, and gastrocnemius muscles, followed by 3 min of potentiation exercises involving jumps and accelerations. After a 3-min rest period, players proceeded to the designated SSG for that session.

The 2v2 format consisted of four 3-min bouts with 2-min recovery periods. The 4v4 format included three 4-min bouts, also interspersed with 2-min recoveries, while the 6v6 format comprised two 6-min bouts with the same recovery duration. Total playing time was comparable across formats (12 min), while inter-bout recovery differed (2v2: 6 min; 4v4: 4 min; 6v6: 2 min), based on specificity of the playing format and typical demands. All matches were played on fields with a small goal centered on the end line and without the offside rule. Field dimensions were 20 × 15 meters (75 m^2^ per player) for 2v2, 28 × 21 meters (74 m^2^ per player) for 4v4, and 35 × 26 meters (76 m^2^ per player) for 6v6. Maintaining a similar relative area per player across formats was intended to control for pitch-size effects and better isolate the effect of player number/format. All formats were conducted without verbal encouragement or tactical instructions from coaches or staff to avoid influencing players’ behavior. Additionally, multiple balls were placed around the perimeter of each field to ensure quick ball repositioning and minimize game interruptions. Player groupings were organized to ensure a mix of playing positions within each match and to maintain balance in skill level and playing performance. Specifically, coaches assisted in forming teams so that each team included a comparable positional distribution (defender/midfielder/forward) and avoided clustering of the highest-performing players in the same unit based on coaches’ season-long performance appraisal. Coaches were consulted to assist in forming these balanced and representative teams.

### 2.4 Procedures and assessments

Assessments were conducted at three time points: at rest (prior to the warm-up), approximately 5 min after the final bout of the SSGs, and again 24 h following the completion of the SSGs, to capture acute (immediate post-session) and short-term recovery (24 h) responses relative to baseline. All evaluations took place in a climate-controlled room, where trained evaluators first measured creatine kinase levels and muscle stiffness, followed by the drop jump test to assess the RSI. The sequence and order of all procedures were kept consistent across all sessions.

#### 2.4.1 Creatine kinase

Following local asepsis of the cubital fossa with alcohol, a small 30-μl blood sample was drawn into a heparinized capillary tube by certified phlebotomists. This small volume was sufficient for the assessment of Creatine Kinase (CK) concentration. The CK level was then determined using reflectance photometry at 37 °C with a Reflotron Plus apparatus (Roche, Germany). CK was analysed as absolute concentration (U/L) at each time point. Baseline values are reported to contextualize inter-individual variability, and post-session differences were interpreted with this variability in mind.

#### 2.4.2 Muscle stiffness

Muscle stiffness was assessed using a handheld myometer (MyotonPRO, Myoton AS, Estonia), a non-invasive device that delivers a brief mechanical impulse to the muscle and records the tissue’s response (27). Measurements were taken with the participant in a relaxed, supine position, with the limbs supported to minimize tension. Stiffness was evaluated in biceps femoris (long head). The biceps femoris was selected because hamstring musculature is frequently involved in high-speed and change-of-direction actions in soccer (28) and is commonly monitored in fatigue-related research, however, we acknowledge that this provides a muscle-specific rather than whole-body index. The probe was placed perpendicularly to the skin surface at standardized anatomical landmarks, previously marked to ensure consistency across time points. Three consecutive measurements were recorded at each site, and the mean value was used for analysis. All assessments were performed by the same trained evaluator to reduce inter-rater variability. Muscle stiffness was measured in newtons per meter (N/m).

#### 2.4.3 Reactive strength index (RSI)

The MyJump 2 app was used in this study to assess the Reactive Strength Index (RSI) of participants. This application is a reliable tool for measuring jump performance, specifically by recording measures such as flight time and contact time (29). The testing procedure involved participants performing drop jumps from a standardized height (50 cm), while ensuring proper landing mechanics (30). Upon landing, participants were required to immediately execute a maximal vertical jump. The MyJump 2 app, utilizing the camera of the mobile phone device, recorded the jump sequence and captured the flight and contact times. The app estimates jump height from flight time (using the standard projectile-motion equation) and calculates RSI as jump height (m) divided by ground contact time (s) (29). Therefore, RSI is expressed in m/s. Players performed three jumps, interspersed with 30 s of rest between each attempt. Before data collection, participants completed a brief familiarization session comprising standardized instruction and practice trials to ensure consistent drop-jump technique. The best attempt was selected for further analysis, with the RSI expressed in meters per second (m/s).

#### 2.4.4 Rating of perceived exertion (RPE)

The Rating of Perceived Exertion (RPE) was employed as a subjective measure of exercise intensity experienced by the participants during the SSGs. The Borg CR10 scale, a widely recognized and validated psychophysical tool, was utilized for this purpose (31). Participants were instructed to verbally report their overall feeling of exertion 5-min immediately following the last SSG bout, using a scale ranging from 0 (“nothing at all”) to 10 (“maximal”) reflecting session intensity perception (CR10 session RPE). All players were familiar with CR10 reporting through routine team training monitoring. This scale allowed individuals to quantify their perceived effort, reporting individually in a dedicated form.

### 2.5 Statistical procedures

To examine the influence of varying game formats (2v2, 4v4, and 6v6) and time points (at rest, immediately post-exercise, and 24 h recovery) on muscle stiffness, creatine kinase and RSI, a two-way repeated measures analysis of variance (ANOVA) was conducted using JASP (version 0.19.3, The Netherlands). This approach was selected due to the within-subject nature of the design, where each participant completed all three game formats and was assessed at each of the three time points. Prior to analysis, the assumption of sphericity was assessed using Mauchly’s test, and where violated, Greenhouse–Geisser corrections was applied to the degrees of freedom. Statistical significance was determined at an alpha level of *p* < 0.05. In the event of significant main effects or interaction effects, pairwise comparisons were performed using Bonferroni post-hoc tests to control for multiple comparisons and identify specific differences between conditions. Partial eta-squared (ηp^2^) was calculated as a measure of effect size to quantify the proportion of variance in the dependent variable(s) attributable to each factor and their interaction. Pearson correlation analysis was used to examine the relationships between RPE scores and muscle stiffness, creatine kinase and RSI, with correlation magnitudes interpreted as small (*r* = 0.1–0.3), moderate (*r* = 0.3–0.5), and large (*r* > 0.5). For each time point, correlations were computed using pooled observations across formats. Data distributions were screened prior to parametric analyses (visual inspection of histograms and Q–Q plots), and linearity was verified for correlation analyses. Pearson correlation was applied for approximately linear relationships. Statistical procedures were conducted for a *p* < 0.05.

## 3 Results

Significant interactions between time and small-sided game format were observed for all dependent variables: muscle stiffness (*p* = 0.001, ηp^2^ = 0.082), creatine kinase (*p* < 0.001, ηp^2^ = 0.975), and RSI (*p* < 0.001, ηp^2^ = 0.193). Table 1 shows the statistics and within-format game comparisons across the different assessment time points.

**Table 1:** Statistics and within-format game comparisons across the different assessment time points.

Variables	Rest	Post-SSG	Post-24 h
2v2 format			
Muscle stiffness (N/m)	236.8 ± 17.5^b^^,^^c^	325.1 ± 16.5^a^	327.4 ± 23.2^a^
Creatine kinase (U/L)	134.4 ± 9.9^b^^,^^c^	306.1 ± 10.4^a^^,^^c^	362.8 ± 9.7^a^^,^^b^
RSI (m/s)	2.34 ± 0.10^b^^,^^c^	1.63 ± 0.12^a^^,^^c^	1.73 ± 0.14
4v4 format			
Muscle stiffness (N/m)	233.0 ± 20.0^b^^,^^c^	317.8 ± 19.0^a^^,^^c^	305.6 ± 22.2^a^^,^^b^
Creatine kinase (U/L)	139.0 ± 7.4^b^^,^^c^	264.1 ± 6.3^a^^,^^c^	310.2 ± 6.3^a^^,^^b^
RSI (m/s)	2.33 ± 0.08^b^^,^^c^	1.74 ± 0.10^a^^,^^c^	1.88 ± 0.12^a^^,^^b^
6v6 format			
Muscle stiffness (N/m)	234.5 ± 18.2^b^^,^^c^	318.6 ± 15.5^a^^,^^c^	307.8 ± 24.9^a^^,^^b^
Creatine kinase (U/L)	135.1 ± 9.1^b^^,^^c^	241.4 ± 6.6^a^^,^^c^	268.0 ± 6.7^a^^,^^b^
RSI (m/s)	2.35 ± 0.08^b^^,^^c^	1.78 ± 0.12^a^^,^^c^	1.98 ± 0.16^a^^,^^b^

RSI, reactive strength index; SSG, small-sided games.

a Significantly different from rest (*p* < 0.05).

b Significantly different from post-SSG (*p* < 0.05).

c Significantly different from post-24 h (*p* < 0.05).

**Figure 1:**
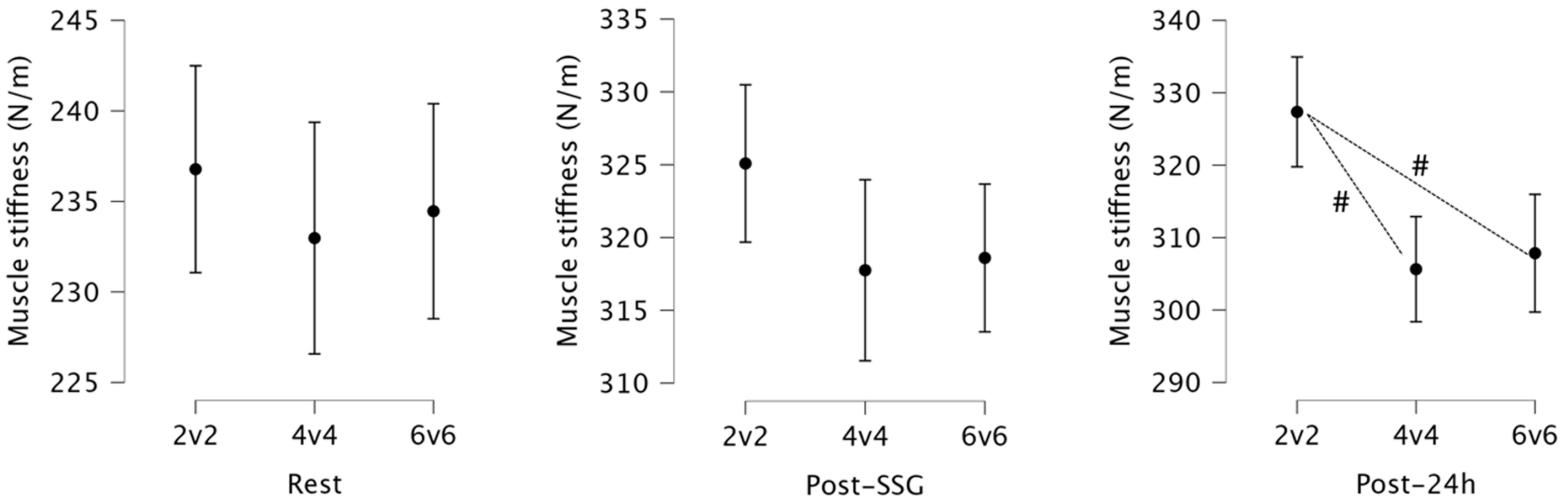
Descriptive statistics for muscle stiffness at rest, immediately post-small-sided games (SSG), and 24 h post-SSG across the three game formats. #: Significant differences between formats (*p* < 0.05).

Simple effects analysis revealed no significant differences in muscle stiffness between the small-sided game formats (2v2, 4v4, 6v6) at rest (*p* = 0.678, ηp^2^ = 0.007) or immediately post-SSG (*p* = 0.143, ηp^2^ = 0.036). However, a statistically significant difference in muscle stiffness between the groups was observed 24 h post-SSG (*p* < 0.001, ηp^2^ = 0.151), indicating that the type of small-sided game played had a significant effect on muscle stiffness during the recovery period. At 24 h post-SSG, significant differences in muscle stiffness emerged between the SSGs formats. Specifically, participants who played 2v2 exhibited significantly higher muscle stiffness compared to those who played 4v4 (Mean Difference = 21.722, *p* < 0.001) and those who played 6v6 (Mean Difference = 19.514, *p* = 0.002). Figure 1 presents the statistics for muscle stiffness and highlights the significant variations between playing formats.

For the rest, there was no statistically significant difference between groups (*p* = 0.066, partial η^2^ = 0.050), indicating only a small effect size. In contrast, the post-SSG and post-24 h showed highly significant differences between groups (*p* < 0.001, partial η^2^ = 0.920; and *p* < 0.001, partial η^2^ = 0.963, respectively), reflecting large effect sizes.

**Figure 2:**
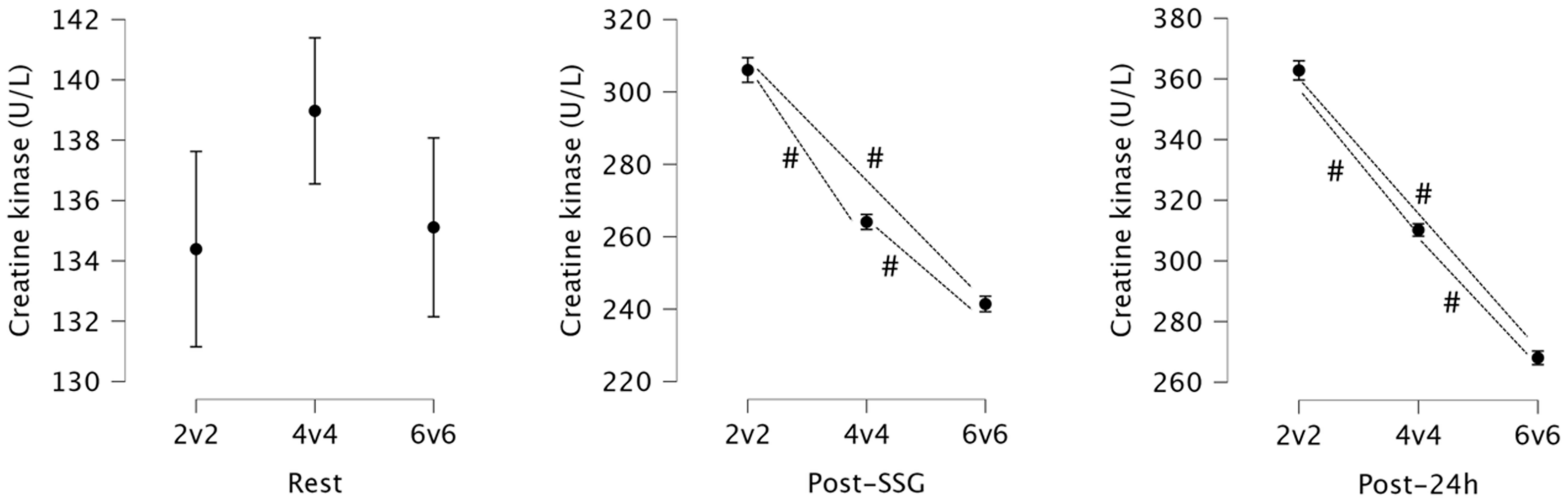
Descriptive statistics for creatine kinase at rest, immediately post-small-sided games (SSG), and 24 h post-SSG across the three game formats. #: Significant differences between formats (*p* < 0.05).

Significant pairwise differences in creatine kinase levels were observed post-SSG and at 24 h post-SSG. Immediately after the SSGs, the 2v2 group showed significantly higher creatine kinase compared to both the 4v4 (Mean Difference = 41.972, *p* < 0.001) and 6v6 groups (Mean Difference = 64.639, *p* < 0.001). Similarly, the 4v4 group had significantly higher creatine kinase than the 6v6 group (Mean Difference = 22.667, *p* < 0.001). These differences persisted and were even more pronounced at 24 h post-SSG, with the 2v2 group exhibiting significantly higher creatine kinase compared to the 4v4 (Mean Difference = 52.611, *p* < 0.001) and 6v6 groups (Mean Difference = 94.778, *p* < 0.001), and the 4v4 group showing significantly higher creatine kinase than the 6v6 group (Mean Difference = 42.167, *p* < 0.001). Figure 2 presents the statistics for creatine kinase and highlights the significant variations between playing formats.

Simple effects analysis of RSI revealed no significant differences between the small-sided game formats (2v2, 4v4, 6v6) at rest (*p* = 0.612, ηp^2^ = 0.009). However, significant differences in RSI between the groups emerged immediately post-SSG (*p* < 0.001, ηp^2^ = 0.249) and persisted at 24 h post-SSG (*p* < 0.001, ηp^2^ = 0.358).

**Figure 3:**
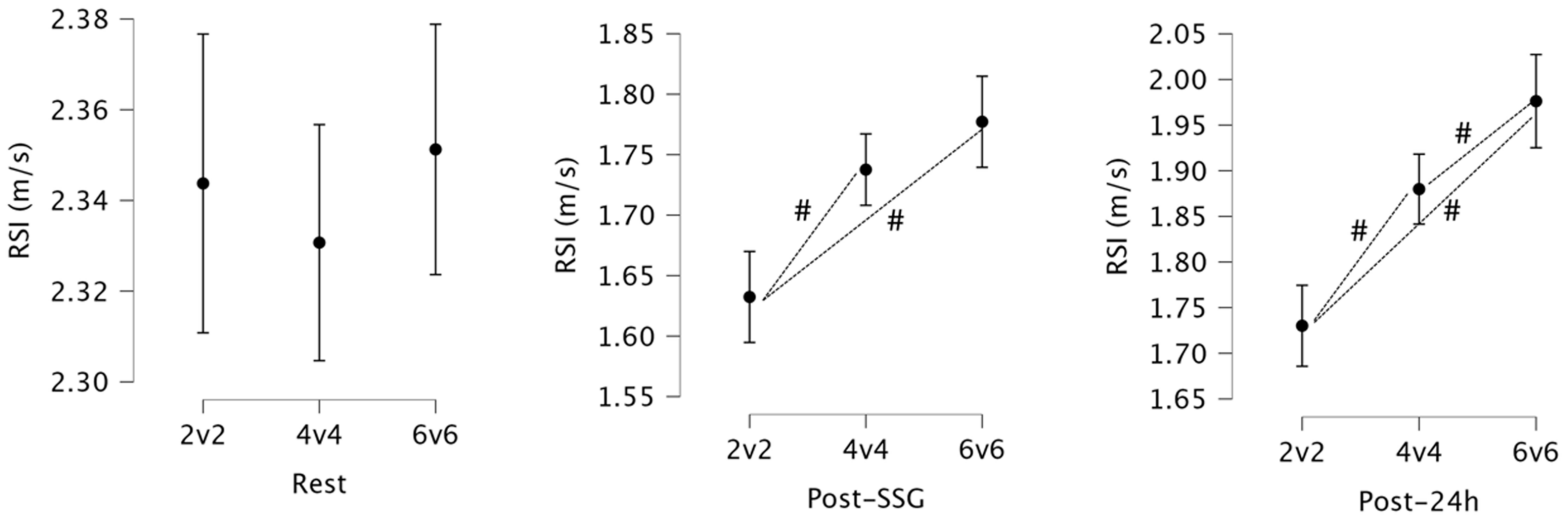
Descriptive statistics for reactive strength index (RSI) at rest, immediately post-small-sided games (SSG), and 24 h post-SSG across the three game formats. #: Significant differences between formats (*p* < 0.05).

Significant pairwise differences in RSI were observed immediately post-SSG and at 24 h post-SSG. Immediately following the games, the 2v2 group exhibited significantly lower RSI compared to both the 4v4 (Mean Difference = 0.105, *p* < 0.001) and 6v6 groups (Mean Difference = 0.145, *p* < 0.001). Similarly, the 4v4 group showed significantly lower RSI compared to the 6v6 group (Mean Difference = 0.040, *p* = 0.044). These differences generally persisted at 24 h post-SSG, with the 2v2 group still showing significantly lower RSI compared to the 4v4 (Mean Difference = 0.150, *p* < 0.001) and 6v6 groups (Mean Difference = 0.246, *p* < 0.001). Additionally, at 24 h, the 4v4 group had significantly lower RSI compared to the 6v6 group (Mean Difference = 0.096, *p* = 0.011). Figure 3 presents the statistics for RSI and highlights the significant variations between playing formats.

**Figure 4:**
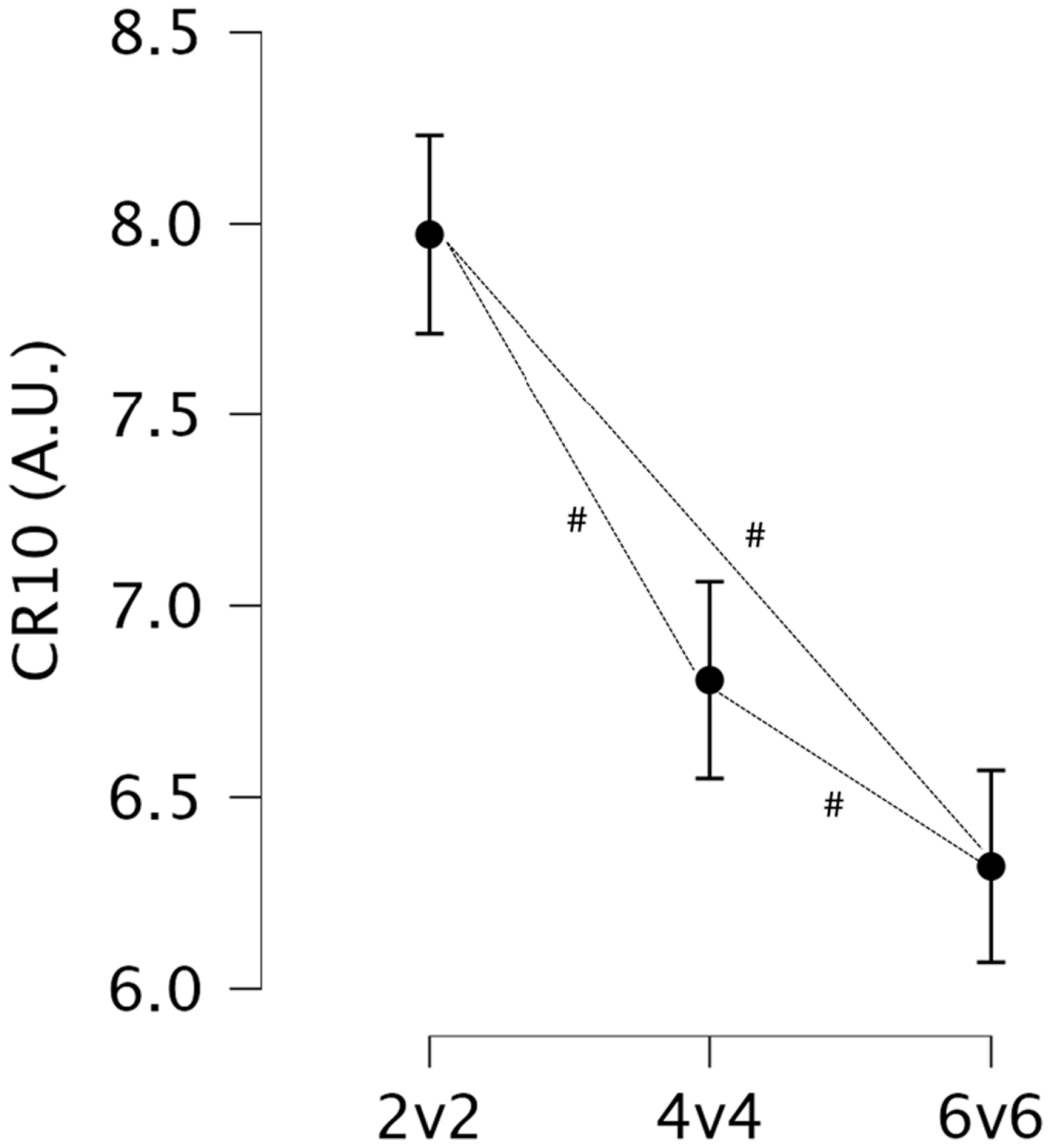
Descriptive statistics for rating of perceived exertion measured using the CR10 Borg’s scale across the three game formats. #: Significant differences between formats (*p* < 0.05).

The ANOVA results revealed a statistically significant difference in RPE between the three small-sided game formats (2v2, 4v4, 6v6), *p* < 0.001. *Post-hoc* analysis with Bonferroni correction revealed significant differences in RPE between all pairs of SSGs formats. Participants in the 2v2 group reported significantly higher RPE compared to both the 4v4 group (Mean Difference = 1.1667, *p* < 0.001) and the 6v6 group (Mean Difference = 1.6528, *p* < 0.001). Additionally, the 4v4 group reported significantly higher RPE compared to the 6v6 group (Mean Difference = 0.4861, *p* = 0.029) (see Figure 4).

**Figure 5:**
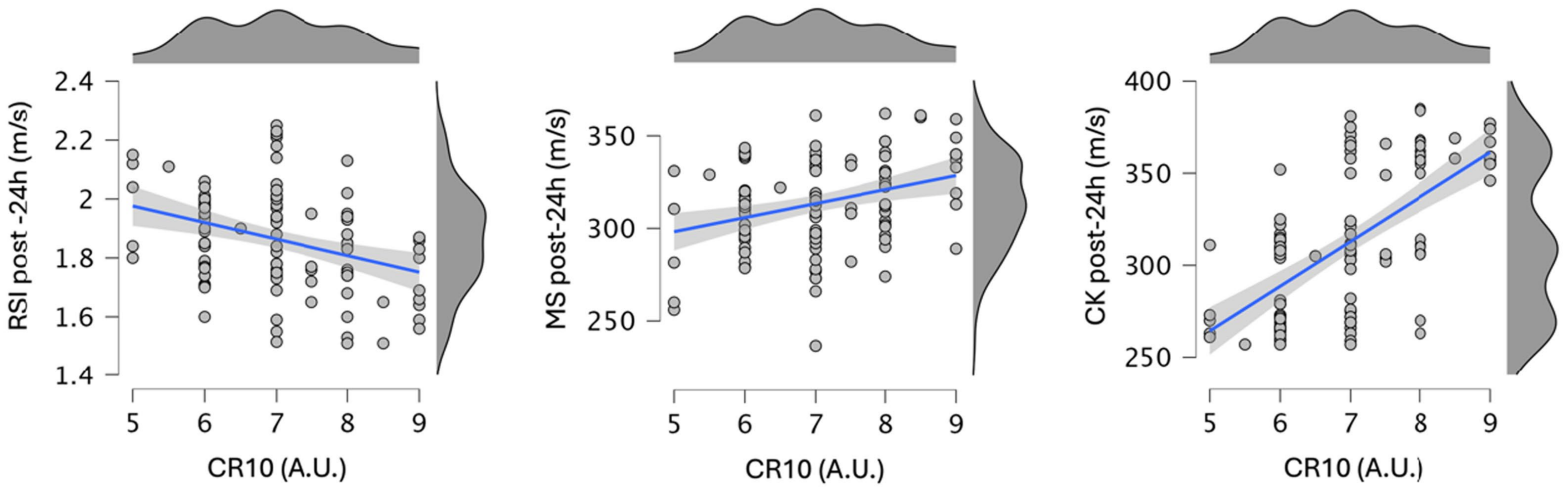
Scatterplot showing the relationships between perceived exertion ratings on the CR10 Borg scale and the values of reactive strength index (RSI), muscle stiffness (MS), and creatine kinase (CK) measured 24 h after the implementation of small-sided games.

Correlations between RPE scores (CR10) and measures of muscle stiffness, creatine kinase, and RSI were assessed post-SSG and 24 h later. Strong correlations were observed between RPE and creatine kinase both post-SSG (*r* = 0.639, 95% CI: 0.510–0.737, *p* < 0.001) and at 24 h post-SSG (*r* = 0.637, 95% CI: 0.507–0.736, *p* < 0.001). Small but significant negative correlations were found between RPE and RSI both immediately after SSG (*r* = −0.210, 95% CI: −0.383 to −0.021, *p* = 0.029) and at 24 h post-SSG (*r* = −0.344, 95% CI: −0.499 to −0.164, *p* < 0.001). A moderate positive correlation was found between RPE and muscle stiffness at 24 h (*r* = 0.314, 95% CI: 0.131–0.266, *p* < 0.001), whereas no significant correlation was observed immediately post-SSG (*r* = 0.082, 95% CI: −0.109 to 0.266, *p* = 0.402). Figure 5 exhibits the scatterplot showing the relationships between perceived exertion ratings on the CR10 Borg scale and the values of RSI, muscle stiffness, and creatine kinase measured 24 h after the implementation of SSGs.

## 4 Discussion

The study revealed significant interactions between time and SSG formats for all variables analyzed (muscle stiffness, creatine kinase, and reactive strength index RSI) underscoring the influence of game format on both immediate and delayed responses to exercise. The 2v2 format consistently elicited greater neuromuscular and biochemical disturbances compared to 4v4 and 6v6 formats, as evidenced by significantly higher post-SSG and 24-h CK levels, increased muscle stiffness at 24 h, and more pronounced reductions in RSI. These results were paralleled by perceptual data, with participants in the 2v2 group reporting significantly higher RPE than those in the other formats. Strong correlations between RPE and CK levels, along with smaller associations with RSI and muscle stiffness, provide exploratory associative context suggesting that higher perceived exertion tends to co-occur with greater short-term perturbations in CK and RSI across conditions. These relationships are not causal and should be interpreted cautiously given the repeated-measures data structure.

Muscle stiffness increased significantly across all SSG formats following exercise, with the most pronounced and prolonged elevations observed in the 2v2 format. While no significant differences between formats were found at rest or immediately post-SSG, muscle stiffness remained significantly higher in the 2v2 group compared to both 4v4 and 6v6 at 24 h post-exercise. Our results, although measured differently, are consistent with a previous study (32) which found that smaller SSG formats induce greater muscle fatigue and higher power-related demands compared to larger formats. Additionally, our findings align with another study (16), which reported that muscle stiffness and recovery kinetics are more pronounced after smaller formats compared to larger ones in soccer players. Because stiffness was assessed only in the biceps femoris, these findings reflect a muscle-specific mechanical response and should not be interpreted as a direct proxy for whole-body neuromuscular status.

Our results suggest that the reduced player number in 2v2 imposes a higher neuromuscular load, which may be related to a higher frequency of high-intensity actions (e.g., accelerations/decelerations and directional changes) and greater individual involvement (33), however, external-load variables were not directly measured in the present study and this mechanistic explanation remains inferential. The sustained elevation in stiffness may reflect altered muscle mechanical behavior after loading (34), but stiffness is not a specific biomarker of microtrauma and can be influenced by muscle tone, fluid shifts/swelling, passive tension, and temperature (19). Therefore, stiffness changes should be interpreted as an indirect indicator of post-exercise muscular status rather than direct evidence of damage (19). Additionally, limited rest between actions in smaller formats may impair the muscle’s capacity to recover (35), contributing to delayed reductions in stiffness. These findings align with previous literature suggesting that exercise formats with higher intensity and reduced recovery periods can lead to greater residual muscle tension and reduced muscle compliance during recovery (16).

Creatine kinase levels were significantly elevated immediately post-SSG and remained high at 24 h post-exercise, with the 2v2 format showing the most substantial increase in CK compared to both 4v4 and 6v6 formats. Specifically, the 2v2 group had markedly higher CK levels than the other formats, both post-SSG and at 24 h, indicating a greater systemic CK response consistent with higher muscle stress and/or membrane perturbation (36, 37), while recognising the substantial inter-individual variability and limited specificity of CK as an isolated marker. Our results align with a previous study (38) which found that muscle damage and inflammation indicators peaked 24 h after a 3 vs. 3 format, suggesting 48 h is needed for recovery. Because CK was not assessed beyond 24 h, the present findings reflect acute and short-term responses only and cannot characterize longer recovery kinetics.

These results may be explained by the increased intensity and mechanical loading associated with smaller-sided games, where players are required to perform more frequent directional changes and accelerations and decelerations (33), within a constrained area. The higher CK response observed in the 2v2 format could also be attributed to the limited number of players, which results in greater individual involvement in each play (39), thus increasing the overall muscular strain. Additionally, the absence of sufficient recovery time between efforts may impair muscle repair processes, leading to prolonged elevations in CK. These findings are consistent with previous research suggesting that SSGs, due to their intensity and reduced rest periods, induce more significant muscle damage and elevate CK levels more than larger-sided formats (16).

The RSI showed significant decreases immediately post-SSG and at 24 h post-exercise, with the 2v2 format showing the lowest RSI values compared to both the 4v4 and 6v6 formats. These differences suggest that the 2v2 format possibly places a greater demand on the stretch-shortening cycle of the muscles, leading to more substantial impairments in explosive strength and reactive performance. A previous study (40) found that main impact on jumping performance from the SSGs training was a decrease in jump height immediately after the training (at 0 h), with a reduction of approximately 3.2 cm. However, this decrease was trivial by 2 h after the session, and then a small-moderate decrease persisted at 24 h (around 2.5 cm) (40). The possible greater frequency of accelerations and decelerations could contribute to a decrease in the efficiency of the stretch-shortening cycle and, subsequently, lower RSI values. Additionally, the higher RPE observed in the 2v2 group supports the notion that these participants experienced greater neuromuscular fatigue, further exacerbating the decline in reactive strength.

The RPE revealed significant differences between the SSGs, with participants in the 2v2 format reporting the highest exertion levels compared to both the 4v4 and 6v6 formats. Specifically, the 2v2 group had a substantially higher RPE than the 4v4 group, which in turn was higher than the 6v6 group. These findings are aligned with previous reports (41). The lower RPE in the 4v4 and 6v6 formats may be attributed to the more distributed workload, allowing for slightly greater recovery between high-intensity efforts. Importantly, the correlations between RPE and physiological markers further support the observed differences: RPE was strongly correlated with creatine kinase both immediately post-SSG and at 24 h post-exercise, suggesting that higher perceived exertion can be linked to greater muscle damage. Additionally, small but significant negative correlations between RPE and RSI both immediately post-SSG and at 24 h post-SSG suggest that as exertion increases, there is a corresponding decline in explosive performance. Furthermore, a moderate positive correlation between RPE and muscle stiffness at 24 h post-SSG indicates that greater perceived exertion is associated with delayed recovery in muscle stiffness. Because multiple observations originate from the same participants (repeated measures), these associations should be interpreted cautiously as exploratory and associative rather than independent.

While our results provides interesting findings into the neuromuscular and biochemical impact of different SSG formats, several limitations should be acknowledged. The sample size was relatively homogenous, limiting generalizability to broader soccer levels, including females, youth, or elite-level players. Additionally, the study focused on short-term responses (up to 24 h post-exercise), which restricts conclusions regarding longer-term recovery and adaptation. The use of indirect markers such as creatine kinase and muscle stiffness, while informative, may not fully capture the complexity of muscle damage or recovery kinetics. Although the SSG sessions were consistently scheduled to take place 48 h after the players’ last official match, we acknowledge that some residual match-related fatigue or muscle damage may have persisted at baseline, and this is considered in the interpretation of week-to-week comparisons. Because the crossover was implemented over three consecutive weeks, the design remains susceptible to period effects (e.g., training adaptation or cumulative fatigue) and carryover effects, particularly for markers such as CK that may remain elevated beyond 24 h. Although counterbalancing reduces systematic order bias, it does not eliminate carryover, therefore, baseline values should be interpreted as potentially influenced by the preceding week’s load. Moreover, although total playing time was comparable across formats, the formats differed in work–rest distribution, which may independently influence fatigue and recovery markers. Despite these limitations, the findings have practical implications for training load management in team sports. Coaches and practitioners should be aware that smaller formats like 2v2 impose higher short-term perturbations in RPE, CK, RSI, and biceps femoris stiffness at 24 h, and should therefore be scheduled with adequate recovery planning when subsequent training quality is prioritized. Therefore, such formats should be used strategically, with adequate recovery periods and monitoring of athlete readiness, particularly when subsequent training quality is a priority. Although injury outcomes were not assessed, these load and recovery considerations may be relevant when planning training microcycles.

## 5 Conclusion

This study highlights the significant impact of SSG formats on both immediate and delayed neuromuscular, and biochemical responses in soccer players. Among the formats tested, the 2v2 SSG elicited the highest CK levels and the largest reductions in RSI, while muscle stiffness was higher at 24 h compared with 4v4 and 6v6. These findings indicate that reducing player numbers likely increases the physiological and mechanical load. Exploratory correlations between RPE and markers such as creatine kinase and RSI provide complementary context and support the value of perceptual measures in monitoring training load. Overall, the results highlight the importance of carefully selecting SSG formats based on training objectives. Smaller formats may be suited to high-intensity loading phases, provided that recovery is planned and athlete readiness is monitored. Future studies should directly test recovery interventions, longer follow-up windows (e.g., 48–72 h), and injury-related outcomes to confirm whether these responses translate into meaningful risk modification in future longitudinal designs.

## Data Availability

The raw data supporting the conclusions of this article will be made available by the authors, without undue reservation.
